# Methionine Synthase Interacts With the Methionine Adenosyl‐Transferase MATα2 and the DNA Methyltransferase DNMT3b in the Nucleus

**DOI:** 10.1002/jimd.70211

**Published:** 2026-06-17

**Authors:** Manon Jeandel, Jean‐Marc Alberto, Okan Baspinar, Aurélie Robert, Natacha Dreumont, Zeinab Alsahly, David Meyre, Jean‐Louis Guéant, David Coelho

**Affiliations:** ^1^ Inserm, UMRS 1256, NGERE—Nutrition, Genetics, and Environmental Risk Exposure University of Lorraine Nancy France; ^2^ Department of Molecular Medicine, Division of Biochemistry, Molecular Biology, and Nutrition University of Lorraine, University Hospital of Nancy (CHRU) Nancy France; ^3^ National Center and Reference Laboratory of Inborn Errors of Metabolism University of Lorraine, University Hospital of Nancy (CHRU) Nancy France

**Keywords:** compartmentalization, DNA methyltransferase, methionine adenosyl‐transferase, methionine synthase, one‐carbon metabolism

## Abstract

Transmethylation reactions, which are crucial for regulating gene expression, require S‐adenosyl‐L‐methionine (SAM) as methyl donor. The substrate for SAM synthesis is methionine, which can be produced by methionine synthase (MS) whose dysfunctions are associated with SAM synthesis alterations despite the presence of methionine in the milieu, suggesting a preferential use of the methionine produced de novo. This highlights the crucial role of MS activity and would imply nuclear import of SAM or MS nuclear localization, allowing protein–protein interactions with the methionine adenosyl‐transferases (MAT) responsible for SAM production. Using subcellular fractions of human cells, biochemical and cellular approaches, including incorporation of ^14^C‐methyltetrahydrofolate, here we provide the experimental evidence of MS localization and activity in the nucleus where it interacts with MATα2, the catalytic subunit of MATII, and the methyltransferase DNMT3b. These results support the idea that spatial compartmentalization of one‐carbon metabolism could play a major role in regulating the epigenome.

## Graphical abstract

This study shows that methionine synthase (MS) interacts with MATα2, responsible for SAM production, and the methyltransferase DNMT3b in the nucleus. These results support the idea that spatial compartmentalization of one‐carbon metabolism could play a major role in regulating the epigenome.

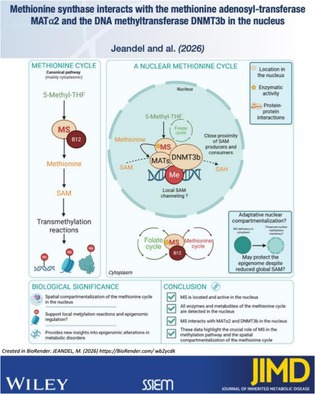

**Abbreviations**
1‐CM: one‐carbon metabolism
5‐methyl‐THF: 5‐methyl‐tetrahydrofolate
AHCY: S‐adenosyl‐L‐homocysteine hydrolase
ATP: adenosine tri‐phosphate
BHMT: betaine‐homocysteine S‐methyltransferase
Cbl: cobalamin
CBS: cystathionine *β*‐synthase
MAT: methionine adenosyl‐transferase
MS: methionine synthase
MSR: methionine synthase reductase
MTHFR: methylene tetrahydrofolate reductase
SAH: S‐adenosyl‐homocysteine
SAM: S‐adenosyl‐L‐methionine
TBP: TATA box binding protein

## 1 Introduction

Regulation of gene expression involves epigenetic modifications that mainly occur in the nucleus or mitochondria for mitochondrial genes [1]. This process requires the dynamic coordination of different regulatory factors compartmentalized within the nuclei [2]. The methylation process requires S‐adenosyl‐L‐methionine (SAM) as the methyl group donor [3]. SAM is synthesized by methionine adenosyl‐transferases (MAT) by transferring the adenosyl group of ATP to methionine during the methionine cycle. Transmethylation reactions also produce S‐adenosyl‐homocysteine (SAH) that is further hydrolyzed by S‐adenosyl‐L‐homocysteine hydrolase (AHCY) to adenosine and homocysteine, which in turn can be degraded in the transsulfuration pathway, via cystathionine *β*‐synthase (CBS), or remethylated by the ubiquitously expressed methionine synthase (MS) encoded by the *MTR* gene. The reaction catalyzed by MS requires the methyl group from 5‐methyl‐tetrahydrofolate (5‐methyl‐THF) produced by methylene tetrahydrofolate reductase (MTHFR) and vitamin B12, also named cobalamin (Cbl), as cofactor. Thus, MS acts at the crossroads between the folate and the methionine cycles in the one‐carbon metabolism (1‐CM) [4, 5]. Alternatively, homocysteine re‐methylation can be catalyzed by betaine‐homocysteine S‐methyltransferase (BHMT), which is primarily expressed in the liver and kidney using betaine as a methyl group donor [6].

The methionine cycle is described as strictly cytosolic, except in some yeasts, where it is restricted to the nucleus [7]. Several enzymes involved in this pathway, BHMT, MAT isozymes, AHCY, and CBS, have been described in the nucleus, suggesting that the BHMT‐dependent methionine cycle could occur in the nuclei of cells expressing BHMT [8, 9, 10, 11, 12]. However, there is no indication that the MS‐dependent canonical methionine cycle could occur in the nuclei of higher eukaryotes.

Nutritional deficit of folate or vitamin B12, or genetic defects of the intracellular metabolism of cobalamin can result in decreased MS activity, leading to the accumulation of homocysteine and the decrease of SAM/SAH ratio reflecting cellular methylation capacity [13]. However, methionine, the substrate required for SAM synthesis, is not only produced by homocysteine re‐methylation but is also provided by diet and protein catabolism [14]. Experimental results in MS‐deficient cells and animal models have shown a reduction in SAM synthesis despite methionine in the cell medium or the animals' diet [15]. These observations raise the question of cellular methionine ‘pools’ that could be preferentially used for SAM synthesis. It also strongly suggests that de novo methionine synthesis is particularly important for SAM production. This hypothesis is based on various data in the literature which show that the use of methionine for the synthesis of SAM, necessary for transmethylation reactions, or incorporation into protein synthesis differs according to the quantity of methionine available and the origin of this methionine, that is dietary intake or homocysteine re‐methylation [16, 17]. Altogether, these data indicate that the partitioning of methionine between its different possible uses—transmethylation and protein synthesis—is a complex process that remains poorly understood. Moreover, it has been shown that under standard dietary conditions, the amount of methionine synthesized de novo by MS represents a minor fraction, approximately 3% of the total cellular methionine pool [18]. The importance of MS activity and SAM needs on cellular proliferation and post‐replicative DNA methylation also support the idea that SAM and its substrate, methionine, should be present in the nuclear compartment. Impaired activity of MS alters cell proliferation through epigenomic mechanisms in tissues with high proliferative index, such as hematopoietic and epithelial cells, with clinical consequences such as megaloblastic anemia, glossitis and intestinal mucosal atrophy [15].

All these recent advances support the concept that the spatial compartmentalization of the methionine cycle could play a major role in the dynamic regulation of the epigenome by increasing the local concentration of related enzymes and substrates. Moreover, since several enzymes involved in 1‐CM have been localized into the nucleus, the idea that all required metabolites, including methionine, are not necessarily transported from the cytoplasm but produced in situ is relevant. Thus, we hypothesized that the crucial role of methionine synthase activity in SAM synthesis suggests a preferential use of de novo‐produced methionine. The local production of metabolites involved in SAM synthesis would imply a nuclear localization of MS and possibly novel protein–protein interactions. Here, we show that all the enzymes of the methionine cycle, including MS, are present in the nuclear compartment of HepG2 cells and fibroblasts from control and *cblG* patients with deficient MS activity. Our study further revealed that MS is also located in the nucleus, where it is active and interacts with MATα2 and DNMT3b methyltransferase. Thus, our work uncovers a novel level of spatial compartmentalization of the enzymes involved in the methionine cycle that could allow a proximity between the producers and consumers of the metabolites involved in the methylation reactions.

## 2 Material and Methods

### 2.1 Cell Lines and Culture Conditions

The patient's fibroblasts were provided by McGill University in Canada (D.R. Rosenblatt; *cblG* 2), the University Hospital of Zurich in Switzerland (M.R. Baumgartner; *cblG* 1), and the National Reference Laboratory (LBMR) of Inborn Errors of Metabolism in the CHRU Hospital of Nancy in France (*cblC* and *cblE*). HepG2 cells (HB‐8065), HEK293T cells (ACS‐4500) and control fibroblasts (PCS‐201‐010) were purchased from LGC Standards (Teddington, United Kingdom).

**Table:** 

Complementation groups	Gene	Mutation	Protein effects
WT	/	/	/
*cblC*	*MMACHC*	c.271dupA c.616C>T	p.R91KfsX14 p.R206W
*cblE*	*MTRR*	c.7A>T	p.R3W
*cblG1*	*MTR*	c.381delA c.2876G>A	p.L128AfsX5 p.V892V
*cblG2*		c.609‐1088G>A	p.A203EfsX13

Fibroblasts were grown in Dulbecco's Modified Eagle Medium high glucose (DMEM) containing 0.03 g/L of methionine (Sigma Aldrich Cat#D6429) supplemented with 10% fetal calf serum (v/v) (Sigma Aldrich Cat#P30‐3306), 1% sodium pyruvate (100 mM) (Gibco Cat#P4333) and 1% (100 U) penicillin–streptomycin (v/v) (Gibco Cat#S8636). The medium was changed twice a week until the cells reached 80% confluence. All cells have been used with a similar number of passages between 10 and 21. HepG2 cells and HEK293T cells were grown in Dulbecco's modified Eagle medium high glucose (DMEM) (Sigma Aldrich Cat#D6429) supplemented with 10% fetal calf serum (FCS) (v/v) (Sigma Aldrich Cat#P30‐3306) and 1% (100U) penicillin–streptomycin (v/v) (Gibco Cat#S8636). Cells were trypsinized every 4 days to harvest cells in the exponential proliferation phase. All cell lines were cultured at 37°C with 5% CO_2_ and were tested (Polymerase Chain Reaction: PCR) negative for mycoplasma contamination.

### 2.2 Preparation of Separate Nuclear and Cytoplasmic Fractions, Protein Total Extraction, and Dosage of Protein Quantity

Nuclear and cytoplasmic fractions were separated using an NE‐PER kit (ThermoFisher Scientific Cat#78833). Briefly, cells were cultured in a T150 flask and were washed two times with 15 mL of phosphate‐buffered saline (1× PBS) (Sigma Aldrich Cat#1408), followed by the addition of trypsin–EDTA (Sigma Aldrich Cat#T3924). Once cells were detached from the flask, 6 mL of FBS was added, and the cells were collected. Following centrifugation at 200 g for 5 min, the cell pellet was washed with 5 mL of 1× PBS and centrifuged under the same conditions before storing at 4°C to realize subcellular fractionation. Subsequently, the cell pellet was resuspended in 1 mL of CER I buffer and incubated for 10 min on ice. Then, 55 μL of CER II buffer was added and incubated for 1 min on ice before centrifugation at 16000 g for 5 min. We have modified the manufacturer's protocol to avoid possible cytoplasmic contamination in nuclear fractions or mitochondrial contamination. We modified the centrifugation speed (10 000 g) to maintain mitochondria in the cytoplasmic fraction. Furthermore, after the cytoplasmic fraction's recovery, we repeated the cytoplasm extraction step to wash the pellet containing the nucleus and cell debris to remove any risk of contamination. After washing the nuclear pellets, 500 μL of NER buffer was added and the pellet was resuspended before the incubation on ice for 40 min. Every 10 min fraction was submitted to Vortex. Nuclear fractions were submitted to a centrifugation at 10000 g for 10 min, and the supernatant was recovered. All fractions were submitted to a WES capillary electrophoresis assay (Bio‐Techne Cat#SM‐W001; Cat#DM‐001; Cat#DM‐002) against *α*‐tubulin (Cell Signaling Cat# 2144) as a cytoplasmic marker and TATA Box Binding Protein (TBP) (Abcam Cat# ab63766) as a nuclear marker to check the good separation of each compartment proteins. Additionally, a WES capillary electrophoresis assay against citrate synthase (Cell Signaling Cat# 14309) as mitochondrial marker was realized to analyze the presence or the absence of mitochondrial contamination in each fraction.

For total protein extraction, cellular pellets were extracted in a lysis buffer (NaCl, Tris pH 8, IGEPAL, EDTA pH 8) supplemented with 10% Protease Inhibitor Cocktail (PIC) (Roche Cat# P8340), 10% phenylmethylsulphonyl fluoride, 10% sodium orthovanadate, and 50% NaBu. The supernatant was collected after 30 min of incubation and 12 000 g centrifugation maintained at 4°C for 10 min. The protein extract was dosed with the BCA assay method (Interchim Cat# UP40840A) using BSA as the standard protein.

### 2.3 Capillary Western Blot (Wes) Analyzes

Per the manufacturer's instructions, capillary electrophoresis analyzes were performed using the ProteinSimple Wes System (ProteinSimple, San Jose, CA, USA). The cytoplasmic and nuclear fractions were mixed with Sample Western Sample Buffer and standards (containing 5× sample buffer, 5× fluorescent standard, and 200 mM DTT) to a final concentration of 0.2 μg/μL, reduced and denatured at 95°C for 5 min. After this denaturation step, the prepared samples, blocking reagent, primary antibodies (1:25 dilution for TATA Box Binding Protein (TBP) and 1:50 dilution for *α*‐tubulin), HRP‐conjugated secondary antibodies, and chemiluminescent substrate were dispensed into designated wells in an assay plate. After plate loading, the separation electrophoresis and immunodetection steps took place in the fully automated capillary system (Compass for SW, Bio‐Techne).

### 2.4 Immunoprecipitation

Whole‐cell lysates and cytoplasmic and nuclear fractions were prepared to obtain 500 μg of proteins in each sample. The different lysates were incubated with 5 μL of Dynabeads Protein G (ThermoFisher Scientific Cat#10004D) for 20 min. After that, each lysate was collected and incubated with 5 μL of antibody of interest (Rabbit polyclonal anti‐*MTR* Proteintech Cat# 25896‐1‐AP; Rabbit polyclonal anti‐MAT2A Novus Cat# NBP1‐28605) for 1 h. The lysates were retrieved and incubated with 25 μL of Dynabeads Protein G at 4°C overnight. Samples were recovered and washed four times with 1× PBS. Fifty microliters of 1% SDS (Sigma Aldrich Cat#L4509) were added with 12 μL of 5× Laemmli buffer. After heating at 95°C for 3 min, the samples were collected and loaded onto SDS‐PAGE gel for electrophoresis separation.

### 2.5 Western Blotting

After immunoprecipitation, the totality of the immunoprecipitated total protein, cytoplasmic, and nuclear fractions were loaded per lane for SDS‐PAGE migration. Depending on the protein's molecular mass, the stacking and the separating gel contained 6%–12% of polyacrylamide, respectively. Proteins were electrotransferred onto PVDF or nitrocellulose membranes (Millipore, Molsheim, France) in Tris buffer containing 39 mM glycine, 0.01% SDS and 20% (vol/vol) absolute ethanol. At room temperature, the membranes were blocked either with 5% non‐fat milk or 5% BSA for 1 h. The membranes were then incubated at 4°C overnight with various primary antibodies, Rabbit polyclonal anti‐*MTR* (Abcam Cat# ab66039) (1:1000), Rabbit polyclonal anti‐*MTRR* (Abcam Cat# ab129159) (1:1000), Mouse monoclonal anti‐MAT1A (Proteintech Cat# 67408–1‐Ig) (1:1000), Rabbit polyclonal anti‐MAT2A (for IF, IP and WB) (Novus Cat# NB110‐94158) (1:1000), Rabbit polyclonal anti‐MAT2B (Proteintech Cat# 15952–1‐AP) (1:1000), Rabbit polyclonal anti‐DNMT3b (Novus Cat# NB300‐516) (1:1000), Mouse monoclonal anti‐MTHFR (Abnova Cat# H00004524‐M03) (1:1000), Rabbit polyclonal anti‐AHCY (Proteintech Cat# 10757–2‐AP) (1:1000) and Rabbit polyclonal anti‐CBS (Abcam Cat# ab135626) (1:1000). Appropriate secondary antibodies conjugated to HRP (anti‐rabbit‐HRP Jackson ImmunoResearch Labs Cat# 711–035‐152 and anti‐mouse‐HRP Jackson ImmunoResearch Labs Cat# 705–035‐147) were used for detection with ECL (Amersham Cat#RPN2236) in iBrigt (ThermoFisher Scientific).

### 2.6 Immunofluorescence

Fibroblasts of patients and HepG2 cells $\left(4*10^{6}\text{cells}\right)$ were grown on slides for 24 h and were rinsed two times with 1× PBS (10× D‐PBS). Then, they were fixed with 4% paraformaldehyde for 10 min, permeabilized with 1× PBS 0.01% Triton for 10 min and blocked with 5% of BSA and 5% of FCS for 1 h. Cells were incubated with various primary antibodies, Rabbit polyclonal anti‐*MTR* (Proteintech Cat# 25896‐1‐AP) (1:200), Rabbit polyclonal anti‐*MTRR* (Abcam Cat# ab235354) (1:100), Mouse monoclonal anti‐MAT1A (Proteintech Cat# 67408–1‐Ig) (1:200), rabbit anti‐MATα2 (1:200), Rabbit polyclonal anti‐MAT2A (Novus Cat# NB110‐94158) (1:100) and Rabbit polyclonal anti‐MAT2B (Proteintech Cat# 15952–1‐AP) (1:200) overnight in a humid chamber at 4°C under a gentle checking. Slides were washed three times in 1× PBS for 5 min, then incubated with the secondary antibody (Alexa Fluor anti‐rabbit 488 (ThermoFisher Scientific Cat# A‐11008) and anti‐mouse 594 (Thermo Fisher Scientific Cat# A‐11005)) for 1 h at room temperature under gentle checking. The nuclei were detected with the fluorescent dye 4, 6‐diamidino‐2‐phenylindole (DAPI) (Thermo Fisher Scientific Cat#D1306) (0.43 mg/mL in PBS). Finally, slides were washed four times in 1×BS and mounted using a mounting medium (Fluoromount‐G Carlsbad, CA, USA ThermoFisher Scientific Cat#00‐4958‐02). The immunostained cells were imaged using a Nikon C2 with three laser lines (405, 488 and 543 nm). The images were obtained with an ×60 oil immersion lens (NIS Elements, Nikon Instruments Inc). The following three sequential acquisition settings with the respective wavelength excitation (ex) and emission (em) detection windows were used to capture the fluorophores and eliminate any crosstalk: for DAPI ex: 405/em: 420–490 nm, for AlexaFluor 488 ex: 488/em: 500–531 nm and for AlexaFluor 543 ex: 543/em: 562–592 nm. The scan speed was set at ¼ frame per second, and the image resolution was 2048 × 2048 pixels. The optical thickness was between 0.30 and 0.40 μm, and the calibration was 0.03 μm/pixel. The images were converted to 2 × 12‐bit TIF images, and image files were subsequently processed with ImageJ software before contrast enhancement was carried out in Photoshop using the same setting for all the images.

### 2.7 Duolink Proximity Ligation Assay

This technique is based on the proximity of two proteins. A distance lower than 40 nm results in a red blob that can be visualized by confocal microscopy. The proximity ligation assay (PLA; Duolink in situ PLA reagents) (Sigma Aldrich Cat#DUO92007‐100RXN) was performed to visualize and quantify interactions according to the manufacturer's instructions. After growing fibroblasts and fixing them with 4% paraformaldehyde for 10 min, permeabilizing with 1× PBS 0.01% Triton for 10 min and blocked with blocking reagent of kit for 1 h, they were incubated with various primary antibodies, Rabbit polyclonal anti‐*MTR* (Proteintech Cat# 25896‐1‐AP) (1:200) and Mouse monoclonal anti‐MAT2A (Novus Cat# NBP1‐28605) (1:100) overnight in a humid chamber at 4°C under gentle checking. A pair of oligonucleotide‐labeled secondary antibodies (PLA probes) was used according to the manufacturer's instructions to bind to the primary antibodies. The PLA signal was assigned using a Nikon C2 confocal microscope. Detection of the nucleus was done with the fluorescent dye 4, 6‐diamidino‐2‐phenylindole (DAPI) (Thermo Fisher Scientific Cat#D1306) (0.43 mg/mL in PBS) and the cytoplasm was done with the fluorescent dye phalloidin (Thermo Fisher Scientific Cat#A12379) (1:150 in water). Finally, slides were washed four times in 1× PBS and mounted using a mounting medium (Fluoromount‐G Carlsbad, CA, USA ThermoFisher Scientific Cat#00‐4958‐02). The images were obtained with an ×60 oil immersion lens (NIS Elements, Nikon Instruments Inc) in the same conditions described in the immunofluorescence staining section. Red blobs were counted with BlopFinder software (Centre for Image Analysis at Uppsala University and the work was supported by the EU FP6 Project ENLIGHT and Olink Bioscience).

### 2.8 EdU Click‐iT

The EdU Click‐iT (ThermoFisher Scientific Cat#C10338) was used to study proliferation. In this dosage, EdU, an analog of thymidine modified, has been incorporated in the DNA during the replication and labeled by fluorescence. Fibroblasts of patients and HepG2 cells were grown on slides for 24 h. The component A was diluted in culture medium and added in cultured cells at 10 μmol/L, and cells were incubated for 2 h at 37°C with 5% CO_2_. After that, cells were fixed with 4% paraformaldehyde for 10 min and permeabilized with 1× PBS 0.01% Triton for 10 min. The 1× Click‐iT buffer additive is prepared according to the manufacturer's recommendation in deionized water (1:1000) and incubated in cells for 30 min at room temperature in the dark. After 1 wash in 1× PBS, cells were blocked with 5% BSA and 5% FCS for 1 h, and we realized the immunofluorescence staining of MS as described previously. Finally, slides were washed four times in 1× PBS and mounted using a mounting medium (Fluoromount‐G Carlsbad, CA, USA ThermoFisher Scientific Cat#00‐4958‐02). The images were obtained with an ×60 oil immersion lens (NIS Elements, Nikon Instruments Inc) in the same conditions as described in the immunofluorescence staining section.

### 2.9 Methionine Synthase Activity: MeTHF Labeling With ^14^C

Determination of MS activity was performed by modifying the radioisotope assay described by Chen [19]. In brief, cell lysates were homogenized at 4°C in 0.5 mL of 0.1 M potassium phosphate buffer (pH 7.2) in the presence of protease inhibitors. After centrifugation (20 000 g, 4°C) for 20 min to remove cell debris, the supernatant was used as a crude extract. Cytoplasmic and nuclear fractions, prepared as previously described, were used in this experiment. For measurement of holoenzyme activity, the reaction mixture contained 250 mM DL‐homocysteine, 25 mM dithiothreitol, 0.05 mM S‐adenosyl‐methionine, 50 μM of hydroxocobalamin, 1 μCi (50 μM) 5‐[^14^C]‐methyl‐THF, crude extract and 0.1 M potassium phosphate buffer in a total volume of 100 μL. The enzyme reaction was carried out under a N_2_ atmosphere at 37°C for 20, 40, and 60 min in the dark and then stopped by heating at 95°C for 5 min. The assay measures the radioactive methionine formed from 5‐[^14^C]‐methyl‐THF (PerkinElmer Cat#NEC846) and homocysteine. The mixture was passed through AG‐1X8 (Cl‐) columns (Bio‐Rad, Marnes‐la‐Coquette, France) to separate 5‐[^14^C]‐methyl‐THF which is not retained in the column and [^14^C]‐methionine, which is retained in the column. After elution of the columns to recover [^14^C]‐methionine, the ^14^C radioactivity was measured by radioactive count. The radiolabeled methionine was measured in a Packard liquid scintillation counter. Enzyme activity was expressed as nmols of methionine produced at 20, 40 and 60 min per mg of protein.

### 2.10 Methionine Synthase Activity: Homocysteine Labeling With D4

For this experiment, 4 nuclear pellets were pooled, and 1 cytoplasmic fraction was used. The 4 nuclear pellets were homogenized at 4°C in 300 μL of PBS in the presence of protease inhibitors. These pellets were mechanically crushed using a syringe and a 26 G needle, with approximately 15 back‐and‐forth movements, and incubated on ice. Two ultrasonic baths of 15 min were realized at 4°C before centrifugation at 12000 g at 4°C for 30 min. The nuclear fraction is recovered in the supernatant. The cytoplasmic and nuclear fractions are submitted to ultrafiltration using ultrafiltration columns (Sigma‐Aldrich ref.: YM10) according to the manufacturer's instructions. The columns were washed 3 times with 500 μL of PBS and centrifuged again to obtain 50 μL of ultrafiltrated nuclear and cytoplasmic fractions. We have added 50 μL of PBS in the presence of protease inhibitors and realized the protein dosage as described previously. After that, a reactional mix was prepared containing 20 mM MgCl_2_, 50 mM KCl, 25 mM dithiothreitol, 25 mM ascorbic acid, 1 mM serine, 1 mM pyridoxal phosphate, 0.1 mM NADPH, 0.1 mM 5‐methyl‐THF, 0.05 mM OH‐Cbl, 1 mM ATP, 0.5 mM D8‐homocystein, crude extract, and 0.1 M potassium phosphate buffer in a total volume of 50 μL. The enzyme reaction was carried out under an N_2_ atmosphere at 37°C for 60 min in the dark and then stopped by adding 5 μL of 1 N acetic acid. Sample's preparation for LCMS/MS was described below.

### 2.11 LC/MS–MS Analysis

Nuclear pellets were solubilized in 1× PBS using mechanical grinding by a 25G needle, followed by an ultrasonic bath for 20 min at 4°C. Then, nuclear fractions in 1× PBS and cytoplasmic fractions were centrifuged at 12000 g for 20 min. The supernatant was collected for LCMS/MS analysis. Briefly, the supernatants were mixed with DTT (Sigma Aldrich Cat#D0632) containing internal standards, precipitated by adding cold methanol and maintained on ice for 30 min. After decanting, the liquid phase was diluted with 4 volumes of 0.1% formic acid. Analysis of 1‐CM intermediates such as methionine, homocysteine, SAM, SAH, cystathionine, and methyl‐THF was performed using an HPLC system coupled to ESI‐Triple quadrupole mass spectrometer (LCMS 8045, Shimadzu, Kyoto, Japan). The chromatography was realized with a Synergi column Fusion‐RP (Phenomenex, 150x4.6 mm, 4 μm) maintained at 40°C and a 0.5 mL/min flow rate of the mobile phase as a mixture of solvent A (0.1% formic acid in water) and B (0.1% formic acid in acetonitrile). Results were analyzed using Insight software V3.1 (Shimadzu, Kyoto, Japan). Samples with concentrations hovering near the detection limits of the instrumentation were excluded to ensure robust results and guarantee reliable concentration detection well within the detection limit. Finally, the total protein amount was measured using the BCA assay method using BSA as standard protein as previously described.

### 2.12 Plasmid *MTR* Transfection in HEK293 Cells

The cDNAs corresponding to MS 144 kDa and MS 124 kDa constructs were generated from the MGC human *MTR* clone (clone Id: 40146647; Horizon) using the CloneAmp HiFi Premix (Takara Cat#639298) following the manufacturer's recommendation. After that, the PCR products were treated with a cloning enhancer (Takara Cat#639298). According to the manufacturer's recommendation, the In‐Fusion Cloning kit (Takara Cat#639298) was used after the treatment. Plasmids were transfected in stellar cells (Takara Cat#636763), and bacteria were cultivated in an LB agar medium with kanamycin. Plasmid DNA extraction was performed with the Plasmid QIAgen Midi prep kit (Qiagen Cat#12145) according to manufacturer recommendations. Plasmids were transfected in HEK293T cells using Jet Pei (Polyplus Cat#101000053). The transfection efficiency was determined by immunofluorescence and confocal microscopy, as described previously.

### 2.13 Quantification and Statistical Analysis

Western blot band intensities were quantified by densitometry using iBright Analysis Software. All errors represent the standard error of the mean (±SEM) from at least three independent experiments except for methionine synthase activity with ^14^C radiolabeled 5‐methyl‐THF, where there are only two independent experiments. A two‐sided *p* < 0.05, 0.01, and 0.001 was considered statistically significant, very significant, and highly significant, respectively. Statistical analyzes were performed using the software GraphPad Prism8.

## 3 Results

### 3.1 Methionine Synthase and the Other Enzymes of the Methionine Cycle Are Localized in the Nucleus

**FIGURE 1:**
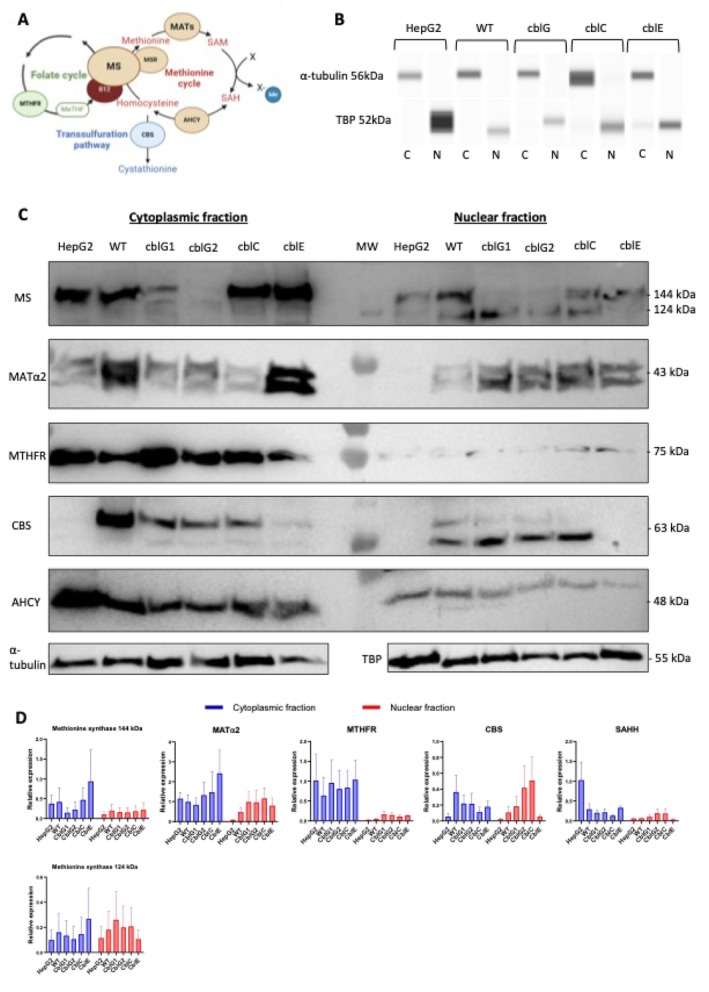
Enzymes of the methionine cycle are also located in the nucleus. (A) Schematic representation of one‐carbon metabolism. AHCY, S‐adenosyl‐L‐homocysteine hydrolase; B12, cobalamin or vitamin B12; CBS, cystathionine *β*‐synthase; MATs, methionine adenosyl transferases; MeTHF, 5‐methyl‐tetrahydrofolate; MS, methionine synthase; MSR, methionine synthase reductase; *MTHFR*, methylene tetrahydrofolate reductase; SAH, S‐adenosyl‐homocysteine; SAM, S‐adenosyl‐L‐methionine; Created with BioRender. (B) Purity of subcellular fractions of HepG2 cells, control, *cblG*, cblC, and *cblE* fibroblasts prepared using the NE‐PERTM nuclear and cytoplasmic extract reagent kit (Pierce). The nuclear and cytoplasmic extracts were subjected to WES capillary electrophoresis assay (ProteinSimple) for *α*‐tubulin (cytoplasmic marker) and TATA Box Binding Protein TBP (nuclear marker) (*N* = 12). (C) Subcellular location of the enzymes involved in the methionine cycle. Nuclear and cytoplasmic extracts were subjected to western blot against *α*‐tubulin, TBP, methionine synthase (MS at 144 or 124 kDa), MATα2, MTHFR, CBS and AHCY. (D) Densitometric analysis of each western blot and statistical analysis; mean of densitometry of protein of interest/reference protein ± SEM (*N* = 3), Normality test and one‐way ANOVA, GraphPad, non‐significative.

Methionine synthase, the enzyme at the crossroad between folate and methionine cycles, is usually described as strictly cytosolic (Figure 1A). Based on the hypothesis that MS could be present in the nucleus we explored this protein's subcellular localization and other proteins involved in the methionine cycle. To investigate this subcellular location, we first realized a subcellular fractionation to separate cytosolic from nuclear proteins of HepG2 cells, that were used as a control for the expression of all MAT subunits, control fibroblasts (WT) and *cblG* patient fibroblasts, which are deficient in MS activity but still present a residual enzymatic activity of the enzyme [20]. We also used here *cblC* and *cblE* fibroblasts, which are respectively deficient in *MMACHC*, a protein which plays a role in intracellular trafficking of cobalamin and in methionine synthase reductase (MSR) which maintains cobalamin in an active state. Both types of genetic defects present a decrease in MS enzymatic activity. The fractionation was realized with HepG2 cells harvested in the exponential phase of proliferation and fibroblasts used at 80% confluence to obtain enough cells to conduct the following experiments. The purity of the different fractions was assessed with a WES capillary electrophoresis assay using *α*‐tubulin as a cytoplasmic marker and TATA Box Binding Protein (TBP) as a nuclear marker. No *α*‐tubulin signal was detected in nuclear fractions, and no TBP signal was detected in cytoplasmic fractions (Figure 1B). This absence of cross‐contamination highlights the purity of our fractions. Furthermore, we have checked the potential contamination with mitochondrial proteins. For this, we performed a WES capillary electrophoresis assay using citrate synthase as mitochondrial marker. Here, we show an absence of this marker in our cytoplasmic and nuclear fractions (Figure S1A,B). For all the experiment using cytoplasmic or nuclear fractions, a WES capillary electrophoresis assay was performed to check the purity of the fractions used.

We then performed immunoblotting of all the enzymes involved in the methionine cycle, including MS, MSR, MTHFR, CBS, AHCY, and MAT subunits in cytoplasmic and nuclear fractions of HepG2 cells, control, *cblG, cblC*, and *cblE* fibroblasts. As expected, we observed a decreased level of MS expression in *cblG* fibroblasts with pathogenic variants of *MTR* compared to control fibroblasts, consistent with the residual activity required for cell viability (Figure 1C,D). Otherwise, MS was present in all the other cell lines. Furthermore, we showed a nuclear location of MS in all cell lines with similar expression levels compared to cytoplasmic fractions. Two bands could be visualized in the MS immunoblot corresponding to the 2 MS splice variants that are differentially expressed according to the proliferation status of the cells, one active form at 144 kDa and one inactive truncated form at 124 kDa (Figure 1C,D). The expression of the active MS is associated with proliferating cells, whereas the expression of the inactive MS isoform is predominant in cells in the stationary phase [21]. We did not observe a significant difference in nuclear MS level in 144 kDa or 124 kDa between *cblG* and WT fibroblasts. This can be explained by the fact that we carried out 3 independent immunoblotting experiments and that it is therefore difficult to obtain close densitometry measurements on 3 independent experiments. Here we have shown that we detect more inactive than active forms of MS in *cblG*. However, the 3 independent immunoblotting experiments have shown the same result with a decrease of the expression of MS at 144 kDa in *cblG* compared to the control. Concerning MSR, we observed a cytoplasmic but also a nuclear localization in all cell lines except in *cblE* fibroblasts carrying genetic mutations in the *MTRR* gene encoding MSR and thus used a negative control for MSR immunoblot (Figures 1A and S1C,D). The following proteins involved in the methionine cycle are methionine and adenosyl‐transferase (MAT) enzymes, responsible for producing SAM. The association of catalytic and regulatory subunits forms these enzymes; MAT I and MAT III enzymes (that are only expressed in the liver) are respectively formed by a dimer or a tetramer of catalytic subunit MATα1, and the MAT II enzyme, whose expression is ubiquitous in the organism, is formed by the catalytic subunit MATα2 and the regulatory subunit MATβ. All MAT subunits are localized in cytoplasmic and nuclear fractions of all cell lines tested (Figures 1C,D and S1A,B). Nevertheless, we are not able to detect a clear blotting of MATβ and MATα2 that can be explained by the highest proportion of MAT I and MAT III enzymes in hepatocytes compared to MAT II [22]. Furthermore, a switch between MATα1 and MATα2 is associated with liver de‐differentiation [23]. These results are consistent with previous studies that have reported nuclear localization of MATα2 [12] and MATα1 [24]. Then, we investigated the subcellular localization of the other enzymes involved in the methionine cycle, including MTHFR, which synthesizes 5‐methyl‐THF, the MS co‐substrate; CBS, which is involved in the transsulfuration pathway to eliminate homocysteine and form cystathionine; and AHCY that hydrolyzes SAH to form homocysteine. Our results confirm the dual cytoplasmic and nuclear locations of AHCY and CBS [8]. Interestingly, we found a slight band corresponding to the presence of MTHFR in the nuclei of HepG2 cells, control, and patient's fibroblasts (Figure 1C,D). Therefore, we provide evidence of the nuclear localization of all the enzymes involved in the canonic methionine cycle.

**FIGURE 2:**
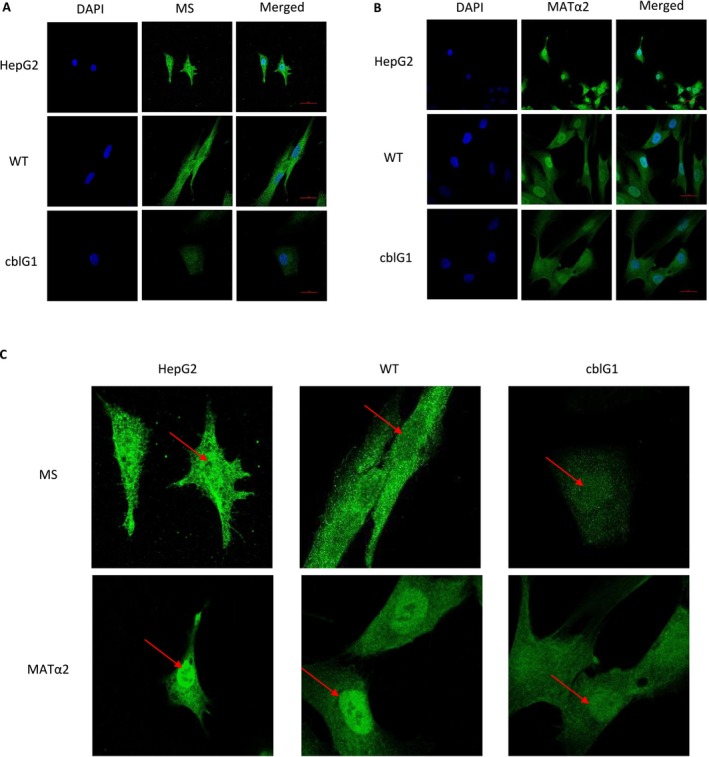
Confirmation of nuclear location of methionine synthase (MS) and MATα2 in HepG2 cells, control and cblG patient fibroblasts. (A, B) Immunofluorescence staining of MS and MATα2 in HepG2 cells, control (WT), and cblG fibroblasts. Cells were stained with anti‐MS (A) and anti‐MATα2 (B) antibodies and visualized by confocal microscopy (*N* = 3). Adjustments of individual colour channels were performed with Photoshop. (C) Zoom of MS and MATα2 staining presented in Figure S2A,B.

To confirm the nuclear localization of these enzymes, we performed immunofluorescent labeling of MS, MSR, and MAT enzymes in HepG2 cells, control, and patient's fibroblasts. Our results, visualized by confocal microscopy in the nucleus plan, indicate a cytoplasmic and nuclear localization of all these proteins in all cells tested (Figures 2A,C and S2A,E).

To further confirm the nuclear localization of MS with a technique that eliminates the need for antibodies and the specific bias they can generate to identify the protein, we produced two plasmids coding for both MS isoforms (144 and 124 kDa) fused with a green fluorescent protein (MS‐GFP). First, we performed an immunofluorescent staining of MS in HEK239T cells to visualize the localization of the endogenous protein in these cells. Our results confirmed in this cell line the cytoplasmic and nuclear localizations of MS (Figure S3A,B). Then, we transfected HEK293T cells with our plasmids and visualized the cells by confocal microscopy (Figure S3C,E). Once again, our results confirmed the dual localization of both MS isoforms in the cytoplasm and nucleus.

Finally, to investigate whether the subcellular localization of MS could differ according to the proliferation status of the cells, we performed immunofluorescent staining of MS using cells incubated with EdU. We did not detect any difference in MS staining between EdU‐labeled and non‐labeled cells, suggesting that cellular cycle status does not influence MS subcellular localization (Figure S3F).

### 3.2 Methionine Synthase Is Active in the Nucleus

**FIGURE 3:**
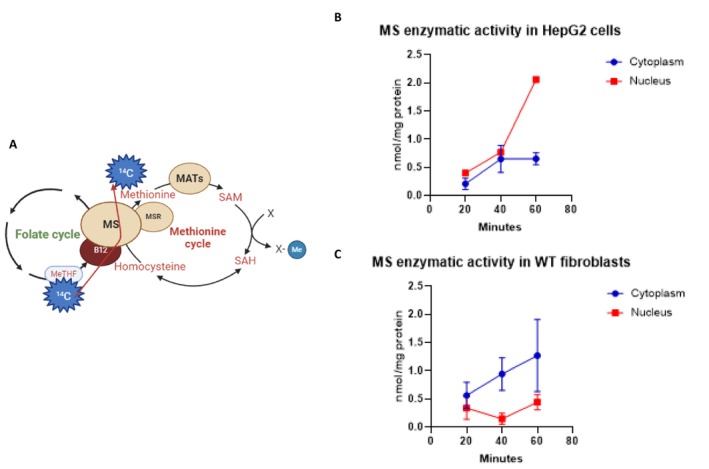
Methionine synthase enzymatic activity in HepG2 cells and control fibroblasts. (A) Schematic representation of incorporation of 5‐[14C]meTHF. (B, C) Kinetic of enzymatic activity of methionine synthase measured after the incorporation of 5‐[14C]‐methyl‐THF in HepG2 cells (A) and control fibroblasts (WT) (B). Cytoplasmic and nuclear fractions were incubated with 5‐[14C]‐methyl‐THF, and [14C]—methionine was measured after 20, 40 and 60 min of incubation; mean ± s.d., *N* = 2.

To further confirm that our results reflect the presence of an active enzyme in the nucleus, we measured MS enzymatic activity in cytosolic and nuclear fractions of HepG2 cells and control fibroblasts (WT). We realized an in vitro incorporation of metabolites radiolabeled using both subcellular fractions incubated with a Master Mix containing 5‐methyl‐tetrahydrofolate radiolabeled with ^14^C in the methyl group (5‐[^14^C]‐methyl‐THF), reducing agents, and all metabolites involved in the MS reaction including cobalamin, the cofactor of the enzyme (Figure 3A). Samples were recovered after 20, 40, and 60 min of incubation to realize an in vitro kinetic of enzymatic activity. Then, liquid chromatography analysis separated 5‐[^14^C]‐methyl‐THF to [^14^C]‐methionine before radioactive counting. Our results revealed the presence of [^14^C]‐methionine in the nuclear fractions of HepG2 cells and WT fibroblasts, demonstrating the presence of active MS in the nucleus (Figure 3B,C). Furthermore, the kinetic enzymatic activity analysis indicated that this activity increased with time, as observed in whole‐cell extracts.

### 3.3 All the Metabolites of the Methionine Cycle Are Present in the Nuclear Fraction

Since the presence of MS in the nucleus would imply a functional methionine cycle in this subcellular compartment, we investigated the presence of the metabolites involved in the methionine cycle by LC/MS–MS in subcellular fractions of HepG2 cells, control fibroblasts (WT), and *cblG* fibroblasts (Figure S4). In fact, the detection of metabolites in the nuclear compartment could show the need for the cell to either bring metabolites into the nucleus as required or to produce metabolites directly where they are needed. These measures allow us to show that methionine cycle metabolites could be present in the nucleus but not that they are produced in this compartment. Indeed, these measurements present some limitations and may also result from a potential leakage of metabolites from the cytoplasm to the nucleus.

We detected methionine in the cytoplasm, as expected, but also in the nuclear fractions with the same order of magnitude. We did not observe any significant difference in the concentration of methionine between *cblG* and control (WT) fibroblasts in both subcellular fractions, which is consistent with the presence of methionine in the cell culture medium (Figure S4A). We detected SAM and SAH in the nuclear fractions of all cell lines, and we did not see a significant difference in SAM levels in the cytoplasmic and nuclear fractions of *cblG* cells compared to WT fibroblasts (Figure S4B,C). Nevertheless, we noticed that the SAM level in the nuclei is much lower than in the cytoplasm (Figure S4B). In addition, we detected cystathionine (CTH) in the nuclear fractions of all cell lines in agreement with previous studies that described a nuclear localization of CBS (Figure S4E). Since we found MTHFR, the enzyme responsible for the synthesis of 5‐methyl‐THF, in the nucleus, we explored the level of this metabolite in both subcellular fractions. Consistently, we detected 5‐methyl‐THF in the nuclei of all cell lines with a concentration approximately 50 times lower than in the cytoplasm. This finding is consistent with MTHFR expression levels detected in western blot experiments, with no significant difference between *cblG* and control fibroblasts (Figure S4F). Collectively, these results highlight the presence of all intermediate metabolites of the methionine cycle in the nuclear fractions of HepG2, control, and *cblG* fibroblasts. Altogether, these measurements of the different metabolites of the methionine cycle are consistent with the expression levels of the related enzymes but do not strictly demonstrate their production in situ.

To investigate if methionine could be directly produced in the nucleus, we realized another experiment of metabolite incorporation modified with stable isotope using deuterated homocysteine with 4 deuterium in carbon skeleton (D4‐Hcy) (Figure S4G). Then, G we measured the labeled methionine (D4‐methionine) by LCMS/MS on cytoplasmic and nuclear fractions of HepG2 cells, control (WT) and *cblG* fibroblasts. For this experiment we performed an in vitro incorporation of D4‐Hcy. We have prepared cytoplasmic and nuclear fractions and checked the purity like it is mentioned before. Measured metabolites were directly produced in cytoplasmic or nuclear fractions. First, we checked the incorporation of D4‐Hcy in subcellular fractions (Figure S4H). We detected D4‐methionine in cytoplasmic and in nuclear fractions of all cell lines. We did not show a significant difference between *cblG* and WT cells, which can be explained by the high concentration of D4‐Hcy used in this experiment that was 10 times higher than MS Km (Figure S4I,J). We were also able to detect D4‐SAH in cytoplasmic and nuclear fractions of all cell lines but not D4‐SAM, potentially because we used high D4‐Hcy concentration which activated preferentially the reversible reaction between homocysteine and SAH. Taken together, these data demonstrate that MS is active in the nucleus to remethylate homocysteine and form methionine directly in situ even though we did not show that D4‐methionine produced in the nucleus was preferentially used for SAM synthesis.

### 3.4 Methionine Synthase Interacts With MAT Subunit

**FIGURE 4:**
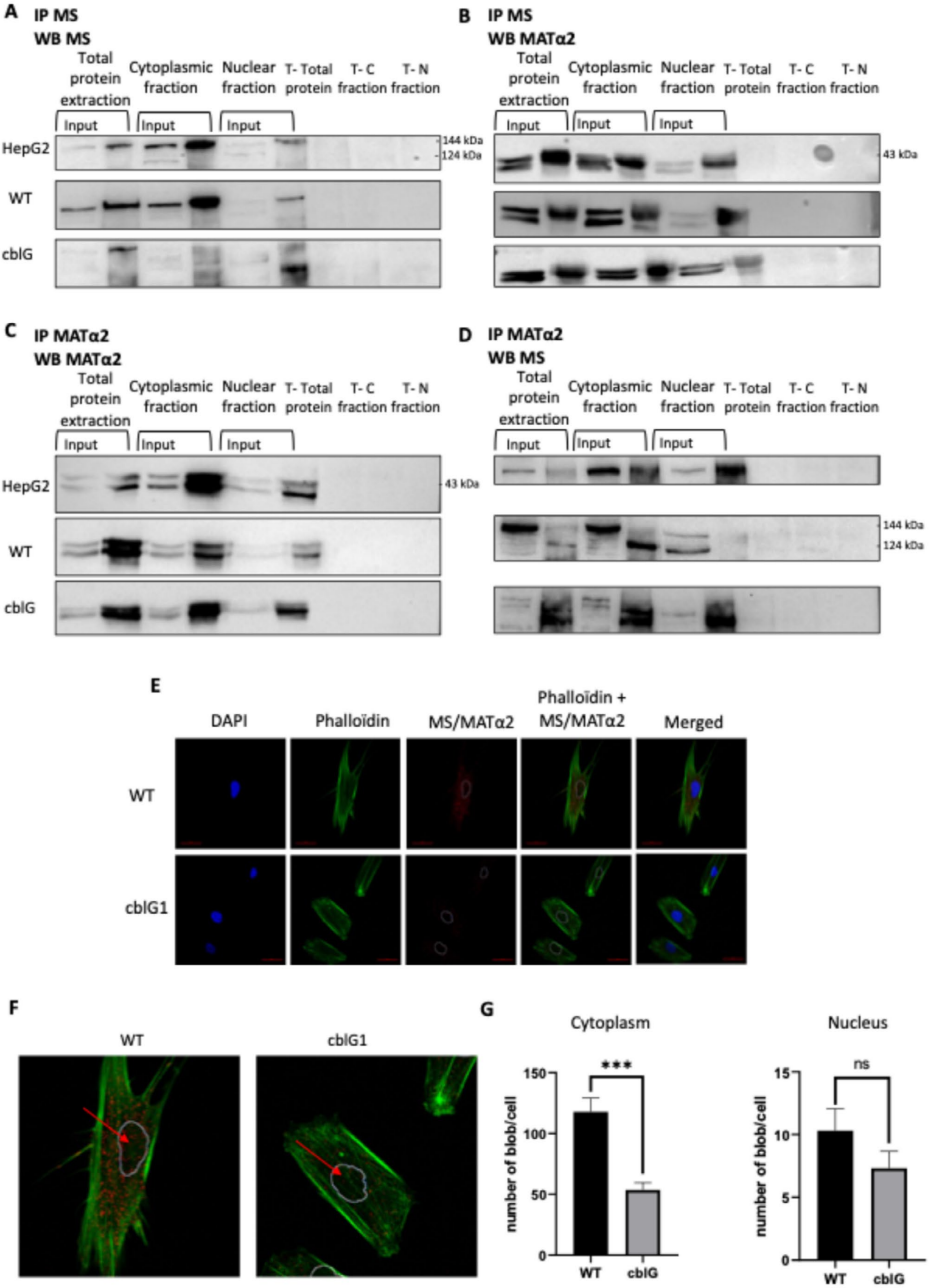
Evidence of protein–protein interaction between methionine synthase and methionine adenosyltransferase 2A (MATα2) in total protein extracts, cytoplasmic and nuclear fractions of HepG2 cells, control and cblG fibroblasts. (A) Validation of methionine synthase (MS) immunoprecipitation. Co‐immunoprecipitation assay was performed on total protein extract, cytoplasmic and nuclear fractions of HepG2 cells, control (WT), and cblG fibroblast cells with an antibody against MS. Immunoprecipitated samples were subjected to western blot against MS with another antibody (*N* = 4). C, cytoplasm; N, nucleus; T, control without antibody which realized immunoprecipitation. (B) Evidence of interaction between MS and MATα2 subunit. Co‐immunoprecipitation assay was performed on total protein extract, cytoplasmic and nuclear fractions of HepG2 cells, control (WT), and cblG fibroblast cells with an antibody against MS. Immunoprecipitated samples were subjected to western blot against MATα2 (*N* = 4). C, cytoplasm; N, nucleus; T, control without antibody which realized immunoprecipitation. (C) Validation of immunoprecipitation of MATα2. Co‐immunoprecipitation assay was performed on total protein extract, cytoplasmic and nuclear fractions of HepG2 cells, control (WT), and cblG fibroblasts cells with an antibody against MATα2. Immunoprecipitated samples were subjected to western blot against MATα2 with another antibody (*N* = 3). C, cytoplasm; N, nucleus; T, control without antibody which realized immunoprecipitation. (D) Validation of interaction between MS and MATα2. Co‐immunoprecipitation assay was performed on total protein extract, cytoplasmic and nuclear fractions of HepG2 cells, control (WT), and cblG fibroblasts cells with an antibody against MATα2. Immunoprecipitated samples were subjected to western blot against MS (*N* = 3). C, cytoplasm; N, nucleus; T, control without antibody which realized immunoprecipitation. (E) Confirmation of interaction between MS and MATα2. Duolink Proximity Ligation Assay was performed on control (WT), and cblG fibroblasts. Red blobs indicate protein–protein interactions between MS and MATα2 (*N* = 3). Adjustments of individual colour channels were performed with Photoshop. (F) Zoom of MS and MATα2 interaction staining presented in Figure S5E. (G) Quantification of the number of blobs in the cytoplasm and in the nuclei of WT and cblG fibroblasts; mean ± SEM, *n* = 3; ***p < 0,001; Two‐way ANOVA; GraphPad.

The presence of MS activity in the nucleus suggests that the nuclear synthesis of methionine could be used to synthesize SAM by providing locally the substrate of MAT enzymes. Thus, we next hypothesized that MS could interact with MAT subunits. Whole protein extracts, cytoplasmic fractions, and nuclear fractions of HepG2 cells, control (WT), and *cblG* fibroblasts were submitted to a series of systematic immunoprecipitations and corresponding western blots using antibodies directed against MS, MATα1, MATα2, and MATβ. First, an immunoprecipitation of MS and a western blot with another antibody against MS were performed to confirm the correct protein immunoprecipitation (Figure 4A). The absence of MS in the input samples of the nuclear fractions of HepG2 cells and control fibroblasts indicated a very low amount of MS in the nucleus. The immunoprecipitation of MS followed by a western blot against the same protein amplified the signal obtained in the nuclear fractions. Furthermore, it confirmed the presence of MS in the nucleus.

Then, immunoprecipitated samples with anti‐MS antibody were subjected to different western blots against MAT subunits (Figures 4B and S5B,C). Our results confirmed the dual localization, cytosolic and nuclear, of MS and MATα2. They also revealed a novel interaction in both subcellular compartments between MS and the catalytic subunit MATα2 by immunoprecipitation of MS and western blot against MATα2 (Figure 4B). Here, we can see that the MS at 144 or at 124 kDa could interact with MATα2. The visualization of a band at 144 or 124 kDa could be explain by the necessity to obtain a lot of cells for different experiments like it is mentioned above. When cells are confluents, we see most of the time a band at 124 kDa. Other investigations will be necessary to know if all MS at 144 and 124 kDa could interact with MATα2 and which domains interact. Furthermore, we have shown two bands for the MATα2 isoform in the input but only one band in the immunoprecipitated sample with MS. MS therefore seems to interact with only one MATα2 isoform but this result should be backed up with more detailed analyzes of interaction between the two proteins. Similarly, total protein extract, cytoplasmic fractions, and nuclear fractions were immunoprecipitated with anti‐MATα2 antibody and subjected to western blot against MATα2 and MS to confirm their interaction. Our results confirm this novel interaction in both subcellular compartments (Figure 4C,D). Immunoblotting of MATβ and MATα1 performed on MS‐immunoprecipitated samples did not show any interaction between MS and MATα1 or between MS and MATβ in HepG2 cells, WT, and *cblG* fibroblasts (Figure S5B,C).

Next, we performed a Duolink Proximity Ligation Assay to confirm the novel interaction between MATα2 and MS and its subcellular localization. Experiments were realized in control (WT) and *cblG* fibroblasts using antibodies against MS and MATα2. Positive signals were obtained in all cell lines, thus confirming the interaction between these two proteins in the cytoplasm and the nucleus (Figure 4E,F) (Figure S5A). Then, we investigated the specificity of our staining by measuring the interaction between MS and MATα2 in a *cblG* cell line carrying *MTR* mutations, resulting in reduced MS expression. Our results showed a significant decrease in the interaction with MATα2 in the cytoplasm of *cblG* fibroblasts (*p* < 0.001) (Figure S4G). Altogether, these results confirmed the subcellular location of MS and MATα2 and their protein–protein interaction in both compartments.

### 3.5 Methionine Synthase and MATα2 Interact With the DNA Methyltransferase DNMT3b

Our results might suggest that MATα2 synthesizes SAM preferentially using the methionine synthesized by MS as substrate. To further investigate the role of the nuclear interaction between MS and MATα2, we investigated their potential interaction with a methyltransferase. Indeed, we hypothesized that MS and MATα2 could interact with an enzyme that uses SAM to methylate nuclear targets such as DNA and histones.

**FIGURE 5:**
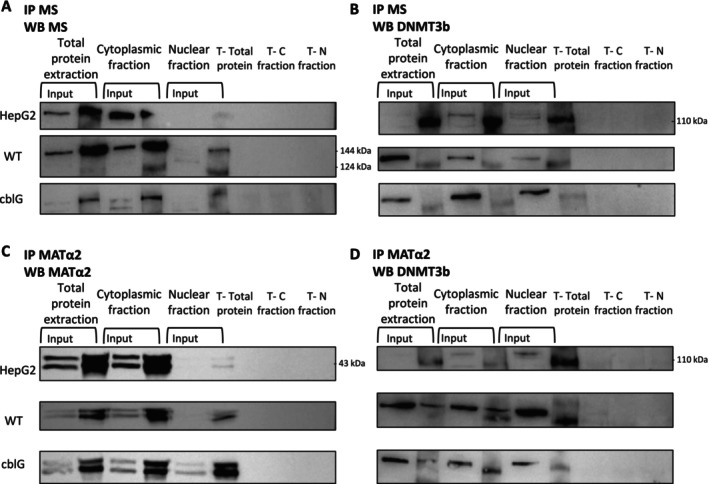
Evidence of protein–protein interaction between methionine synthase (MS), methionine adenosyl‐transferase 2A (MATα2), and DNA methyltransferase 3b (DNMT3b) in total protein extracts, cytoplasmic, and nuclear fractions of HepG2 cells, control, and cblG fibroblasts. (A) Validation of immunoprecipitation of MS in total protein extract, cytoplasmic, and nuclear fractions of HepG2 cells, control (WT), and cblG fibroblasts as described in Figure 5A (*N* = 4). C, cytoplasm; N, nucleus; T, control without antibody which realized immunoprecipitation. (B) Evidence of interaction between MS and DNMT3b. Co‐immunoprecipitation assay was performed on total protein extract, cytoplasmic, and nuclear fractions of HepG2 cells, control (WT), and cblG fibroblasts with an antibody against methionine synthase (MS). Immunoprecipitated samples were subjected to western blot against DNMT3b (*N* = 3). C, cytoplasm; N, nucleus; T, control without antibody which realized immunoprecipitation. (C) Validation of immunoprecipitation of MATα2 in total protein extract, cytoplasmic, and nuclear fractions of HepG2 cells, control (WT), and cblG fibroblasts as described in Figure 5C (*N* = 3). C, cytoplasm; N, nucleus; T, control without antibody which realized immunoprecipitation. (D) Validation of interaction between MATα2 and DNMT3b. Co‐immunoprecipitation assay was performed on total protein extract, cytoplasmic, and nuclear fractions of HepG2 cells, control (WT), and cblG fibroblasts with antibody against MATα2. Immunoprecipitated samples were subjected to western blot against DNMT3b (*N* = 3). C, cytoplasm; N, nucleus; T, control without antibody which realized immunoprecipitation.

We subjected whole cellular, cytoplasmic, and nuclear fractions of HepG2 cells, control (WT), and *cblG* fibroblasts to an immunoprecipitation of MS followed by a western blot with another antibody against MS to confirm the proper immunoprecipitation of the protein (Figure 5A). Then, we further investigated the potential interaction between MS and the methyltransferase DNMT3b, which catalyzes the de novo methylation of DNA, and DNMT1, the DNA methyltransferase needed for maintaining the post‐replicative methylation pattern of DNA. The co‐immunoprecipitation of MS followed by a western blot against DNMT1 in total protein extract, cytoplasmic, and nuclear fractions did not evidence any interaction between MS and DNMT1 (Figure S5D,E). Then, immunoprecipitated samples with MS antibody were subjected to a western blot against DNMT3b. Our results revealed a novel interaction between MS and DNMT3b in total protein extracts and both subcellular fractions [25, 26] (Figure 5B).

We then investigated a potential interaction between MATα2 and DNMT3b. Total protein extracts, cytoplasmic and nuclear fractions were immunoprecipitated with anti‐MATα2 antibody and subjected to western blot analysis for DNMT3b (Figure 5C,D). Our results confirmed the nuclear localization of MATα2 and indicated that MATα2 and DNMT3b interact in the cytoplasmic and nuclear fractions. Altogether, our results suggest that the interaction between MATα2 and DNMT3b might allow the production of SAM where the methyl group is needed for de novo methylation of DNA. Furthermore, it is tempting to speculate that the interactions between MS and MATα2, and between MS and DNMT3b in the nucleus, could contribute to a substrate channeling of both methionine and SAM in the interactome formed by these three enzymes, hence coupling producer and consumer of SAM in the nucleus (Figure 5).

## 4 Discussion

Here, we show that MS, usually described as strictly cytosolic, is also located in the nucleus of human cells where it interacts with MATα2, the catalytic subunit of the MATII enzyme that produces SAM, and with the DNA methyltransferase DNMT3b that uses SAM to methylate de novo DNA. Furthermore, we detected all the enzymes and related metabolites of the methionine cycle in the nuclear compartment, including MTHFR and its catalytic product 5‐methyl‐THF, the co‐substrate of MS. Thus, we propose that the nuclear methionine cycle and these protein–protein interactions could contribute to bring closer SAM producers and consumers to potentially improve the global efficiency of the nuclear methylation reactions.

The presence of all intermediate metabolites of the methionine cycle in the nucleus suggests either the nuclear import of the different enzymes involved in this pathway or the import of metabolites from the cytoplasm to the nucleus (Figure S4). Since the nuclear membrane is most likely permeable to small molecules, this result does not definitely exclude the possibility that the metabolites detected in the nuclear fraction could have diffused through the nuclear pores during the fractionation process, even though the absence of cytoplasmic contamination in the nuclear fractions was verified by WES experiments. Thus, our metabolomic data support the idea that the methionine cycle could occur in the nucleus. Furthermore, many studies indicate that the metabolic enzymes in the nucleus may influence the regulation of epigenomic modifications, such as histone acetylation, by providing locally the metabolites required for this reaction [27, 28, 29]. Subcellular compartmentalization of folate metabolism has already been described with numerous studies highlighting the complex network of interconnected pathways divided into mitochondria, cytoplasm, and nucleus [30]. During folate deficiency, nuclear levels of folate remain high following the nuclear translocation of MTHFD1, which can produce methylenetetrahydrofolate from formate [31]. Similarly, glycine N‐methyltransferase (GNMT) is a cytosolic enzyme imported into the nucleus following folate deficiency [32].

Prior studies have demonstrated that several enzymes involved in the methionine cycle can be found in the nucleus. The presence of MAT enzymes in the nucleus has been described in several cell types [12]. Since transmethylation reactions produce SAH, a potent inhibitor of methyltransferases, the presence of AHCY, the only enzyme able to hydrolyze SAH to homocysteine and adenosine, is required in all subcellular compartments where transmethylation reactions occur. AHCY has been found in the nuclei of various cell types [8, 11, 33, 34, 35]. Furthermore, CBS, which converts homocysteine to cystathionine, can be present in the nucleus [36] [8]. It suggests that the methylation reactions in the nucleus require the presence of 1‐CM enzymes to ensure dynamic control of the needs in SAM. Here, we show that MTHFR is also present in the nuclear fraction (Figure 1). Previous data describing the nuclear localization of the other enzymes of the folate cycle, such as DHFR, SHMT1, SHMT2a, and TYMS, suggest the nuclear production of one‐carbon precursors of methyl groups [37]. Thus, in addition to the presence of MS co‐substrate, our finding that MS and MSR are present in the nuclear compartment supports the idea that the whole methionine cycle can occur within the nucleus (Figures 1 and S1).

We analyzed the sequence of the *MTR* gene using different prediction software, and we did not find any known nuclear localization signal (NLS). Usually, proteins with a molecular mass higher than 40 kDa require a NLS to translocate into the nucleus [38]. However, nuclear translocation of BHMT, MTHFD1 and GNMT occurs despite the absence of a consensus sequence for nuclear localization [10]. Furthermore, we investigated in silico the potential interactions between MS and other proteins using different databases (e.g., FpClass, BioGRID, BioPlex). These databases suggest that 59 proteins could interact with MS, with 33 exclusively nuclear proteins, including a putative methyltransferase (NSUN5P1) and a protein involved in the reorganization of chromatin and regulation of H3K26 methylation (RCCD1). This finding is consistent with our results, indicating a nuclear localization of MS and showing the relevance of looking for additional nuclear partners. While MS expression is lower in the cytoplasm of *cblG* cells compared to control, we observed in the nuclei of *cblG* cells similar expression levels of MS and D4‐methionine in condition of methionine in the culture medium. These data could suggest that the nuclear compartmentalization of the methionine cycle may be regulated to protect the nuclear machinery from the consequences of reduced MS activity, but further experiments will be required to investigate this hypothesis. However, this potential adaptive mechanism is consistent with the absence of modification in global DNA methylation in the brain and eye tissues of transgenic mice with selective MS deficiency despite the global decrease of SAM concentration [39].

Our hypothesis of preferential use of the methionine synthesized de novo for the production of SAM suggested a close interaction between MS and one of the MAT enzymes. Here, we show that MS can interact with MATα2 in the cytoplasm and the nucleus (Figure 4). Although the crucial role of histones and DNA methylation in epigenomic processes is well described, understanding how SAM is provided to nuclear methyltransferases is less documented. Several studies have shown that the nuclear localization of MAT enzymes and protein–protein interactions with methyltransferases contribute to regulating gene expression by providing SAM locally to nuclear enzymes [40]. For instance, a multi‐protein complex SAM‐integrating transcription repression (SAMIT) was proposed to include MAT to produce SAM locally to repress gene transcription through DNA methylation [12, 41]. The interaction between the SAMIT and methyltransferases (MT) suggests that SAM synthesis can be directly coupled to the methylation of target proteins or DNA [12]. Another interaction between MATα2 and a nuclear methyltransferase was reported with the H3K9 methyltransferase SETDB1 to regulate *Ptgs2* expression [42]. Since MATα2 has already been described in the nucleus to interact with various methyltransferases, we postulated that MS could also interact with a MT. Our results confirmed this hypothesis by showing protein–protein interactions between MS, MATα2, and DNMT3b within the nucleus (Figure 5).

## 5 Conclusion

Overall, by showing that MS could interact not only with MATα2 but also with DNMT3b within the nucleus, our results highlight the crucial role of MS in the methylation pathway and the spatial compartmentalization of the methionine cycle. Thus, our study opens new perspectives in understanding the methylation process involved in the nucleus and provides a new comprehension in epigenomic alterations involved in metabolic disorders.

## 6 Limitations

In our study, we did not investigate the molecular mechanisms responsible for the translocation of MS in the nucleus. To address this question, a detailed proteomics analysis of subcellular fractions needs to be performed, including detailed studies about the components of the nucleopore and their post‐translational modifications. Furthermore, even if we did not detect any difference in MS localization according to the proliferation status of the cells used, the hypothesis of a dynamic regulation of the nuclear subcellular localization of MS according to the nucleus should be tested. Moreover, while we provided experimental evidence of MS activity in the nuclear fraction, associated with protein–protein interactions between the producer and a user of SAM, we did not demonstrate that the methionine synthetized de novo in the nucleus is directly used for the local synthesis of SAM. Here, we investigated the interaction between MS and a DNA methyltransferase, but further experiments will be required to investigate the interactions between MS and histone methyltransferases, including SETDB1 that interacts with MATα2. Finally, further experiments will also be needed to characterize the interaction detected between MS and DNMT3b and to investigate whether MATα2 is required.

## Author Contributions

**Manon Jeandel:** conceptualization, methodology, formal analysis, investigation, writing – review and editing, visualization. **Jean‐Marc Alberto:** formal analysis, investigation. **Okan Baspinar:** formal analysis, investigation. **Aurélie Robert:** investigation. **Natacha Dreumont:** formal analysis, methodology, investigation. **Zeinab Alsahly:** investigation. **David Meyre:** resources, writing – review and editing. **Jean‐Louis Guéant:** conceptualization, resources, writing – review and editing, supervision, project administration. **David Coelho:** conceptualization, resources, writing – review and editing, supervision, project administration.

## Funding

This work was supported by grants from the Biology‐Medicine‐Health department of the University of Lorraine, FHU ARRIMAGE, the Minister for Higher Education, Research and Innovation (MESRI). The LC/MS–MS acquisition was co‐funded by the European Union through the European Regional Development Fund “FEDER‐FSE” ‘Lorraine et Massif des Vosges’.

## Ethics Statement

The authors have nothing to report.

## Consent

Informed consent was obtained from all individual participants or from their parents included in the study.

## Conflicts of Interest

The authors declare no conflicts of interest.

## Supporting information


**Figure S1:** Nuclear location of other 1CM enzymes in HepG2 cells, control and patient fibroblasts. (A) Absence of mitochondrial contamination in cytoplasmic and nuclear extracts.
The Ne‐PER nuclear and cytoplasmic extract reagent kit was used in fresh cellular pellet of control fibroblasts cells prepared according to the instruction of fabricant (Pierce). The nuclear and cytoplasmic extracts were subjected to WES assay (ProteinSimple) to vinculine (cytoplasmic marker), lamine 1 and 2 (nuclear marker) and citrate synthase (mitochondrial marker) to verify the efficacity of the kit and the absence of cross‐contamination and mitochondrial contamination. (B) Verification of interaction between MS and MATα2 subunit after modification of fractionation protocol to eliminate mitochondrial contamination in cytoplasmic and nuclear fractions. Co‐immunoprecipitation assay was performed on cytoplasmic and nuclear fractions of control (WT) fibroblast cells with an antibody against MS. Immunoprecipitated samples were subjected to western blot against MATα2. C, cytoplasm and N, nucleus. (C) Subcellular location of the enzymes involved in the methionine cycle. The nuclear and cytoplasmic extracts were subjected to western blot against *α*‐tubulin, TBP, MSR, MATα1 and MATβ (*N* = 3). (D) Densitometric analysis of each blot and statistical analysis; mean of densitometry of protein of interest/reference protein ± SEM (*N* = 3), normality test and one‐way ANOVA, GraphPad, non‐significative.

**Figure S2:** Confirmation of nuclear location of methionine synthase reductase (MSR), MATα1, and MATβ in HepG2 cells, control and patient fibroblasts. (A, B) Immunofluorescence staining of methionine synthase reductase (MSR) and MATβ in HepG2 cells, control (WT), and cblG fibroblasts. Cells were stained with anti‐MSR (A) and anti‐MATβ (B) antibodies (*N* = 3). Adjustments of individual colour channels were performed with Photoshop. (C) Immunofluorescence staining of MATα1 in HepG2 cells, control (WT), and cblG fibroblasts. Cells were stained with anti‐ MATα1 antibody (*N* = 3). Adjustments of individual colour channels were performed with Photoshop. (D, E) Control of immunofluorescence staining; AlexaFluor 488: Green staining, and AlexaFluor 594: Red staining. (F) Zoom of MSR, MATβ, and MATα1 staining presented in (A–C).

**Figure S3:** Nuclear location of MS and MS‐GFP plasmids il HEK293T cells and explication of nuclear MS location. (A) Control of immunofluorescence staining; AlexaFluor 488: Green staining. (B) Immunofluorescence staining of MS in HEK293T. Cells were stained with anti‐MS antibody and visualized by confocal microscopy (*N* = 3). Adjustments of individual colour channels were performed with Photoshop. (C) Expression of methionine synthase isoforms (124 kDa and 144 kDa) fused with green fluorescent protein (GFP) in HEK293T cells visualized by confocal microscopy (*N* = 3). Adjustments of individual colour channels were performed with Photoshop. (D) Control of immunofluorescence staining in HEK293T cells transfected with empty plasmid. (E) Zoom of methionine synthase isoforms (124 kDa and 144 kDa) fused with green fluorescent protein (GFP) in HEK293T cells presented in (C). (F) EdU Click‐iTTM staining with immunofluorescence staining of MS in HepG2 cells, control (WT) and cblG fibroblasts. Cells were stained with EdU and anti‐MS antibody and visualized by confocal microscopy (*N* = 3).

**Figure S4:** Evidence of one‐carbon metabolism in cytoplasmic and nuclear fractions of HepG2 cells, control and cblG fibroblasts. (A–F) Metabolites of one‐carbon metabolism were measured by LC/MS–MS in cytoplasmic and nuclear fractions of HepG2 cells, control (WT) and cblG fibroblasts; methionine (A), S‐adenosyl‐L‐methionine (SAM) (B), S‐adenosyl‐homocysteine (SAH) (C), homocysteine (D), cystathionine (CTH) (E), and 5‐methyl‐tetrahydrofolate (5‐methyl‐THF) (F); Mean ± SEM., *n* = 3; **p < 0.01; ***p < 0.001; Two‐way ANOVA; GraphPad. (G) Schematic representation of incorporation of D4‐homocystein. (H–J) Measurement of D4‐homocystein (H), D4‐methionine (I), D4‐SAH (J) after in vitro incorporation of D4‐homocystein in cytoplasmic and nuclear fractions of HepG2 cells, control (WT) and cblG fibroblasts.

**Figure S5:** Evidence of absence of protein–protein interaction between methionine synthase (MS) and DNA methyltransferase 1 (DNMT1) in total protein extracts, cytoplasmic and nuclear fractions of HepG2 cells, control, and cblG fibroblasts. (A) Control of Duolink Proximity Ligation Assay; T‐1: Without primary antibodies and without PLA probes; T‐2: Without primary antibodies with PLA probes, T‐3: With anti‐MS antibody and with PLA probes; T‐4: With anti‐MATα2 antibody and with PLA probes. (B) Absence of interaction between MS and MATβ. Co‐immunoprecipitation assay was performed on HepG2 cells, control (WT), and cblG fibroblasts with antibody against methionine synthase (MS). Immunoprecipitated samples were subjected to western blot against MATβ (*N* = 4). C, cytoplasm; N, nucleus; T, control without antibody which realized immunoprecipitation; (C) Absence of interaction between MS and MATα1. Co‐immunoprecipitation assay was performed on HepG2 cells with antibody against methionine synthase (MS). Immunoprecipitated samples were subjected to western blot against MATα1 (*N* = 4). T‐: control without antibody which realized immunoprecipitation; C, cytoplasm and N, nucleus. (D) Validation of immunoprecipitation of MS in total protein extract, cytoplasmic and nuclear fractions of HepG2 cells and control (WT) fibroblasts as described in Figure 5A (*N* = 4). C, cytoplasm; N, nucleus; T, control without antibody which realized immunoprecipitation; (E) Absence of interaction between MS and DNMT1. Co‐immunoprecipitation assay was performed on total protein extract, cytoplasmic and nuclear fractions of HepG2 cells and control (WT) fibroblast cells with an antibody against methionine synthase (MS). Immunoprecipitated samples were subjected to western blot against DNMT1 (*N* = 3). C, cytoplasm; N, nucleus; T, control without antibody which realized immunoprecipitation.

## Data Availability

The data that supports the findings of this study are available in the Supporting Information of this article.
