# Supplementary material for: Methionine Synthase Interacts With the Methionine Adenosyl‐Transferase MATα2 and the DNA Methyltransferase DNMT3b in the Nucleus
Source: J Inherit Metab Dis. 2026 Jun 17;49(4):e70211. doi: 10.1002/jimd.70211 (PMC13275206; doi:10.1002/jimd.70211)
Supplement: Supplementary file 2 — Figure S2: Confirmation of nuclear location of methionine synthase reductase (MSR), MATα1, and MATβ in HepG2 cells, control and patient fibroblasts. (A, B) Immunofluorescence staining of methionine synthase reductase (MSR) and MATβ in HepG2 cells, control (WT), and cblG fibroblasts. Cells were stained with anti‐MSR (A) and anti‐MATβ (B) antibodies (N = 3). Adjustments of individual colour channels were performed with Photoshop. (C) Immunofluorescence staining of MATα1 in HepG2 cells, control (WT), and cblG fibroblasts. Cells were stained with anti‐ MATα1 antibody (N = 3). Adjustments of individual colour channels were performed with Photoshop. (D, E) Control of immunofluorescence staining; AlexaFluor 488: Green staining, and AlexaFluor 594: Red staining. (F) Zoom of MSR, MATβ, and MATα1 staining presented in (A–C). [file JIMD-49-0-s003.pptx]

## Slide 1
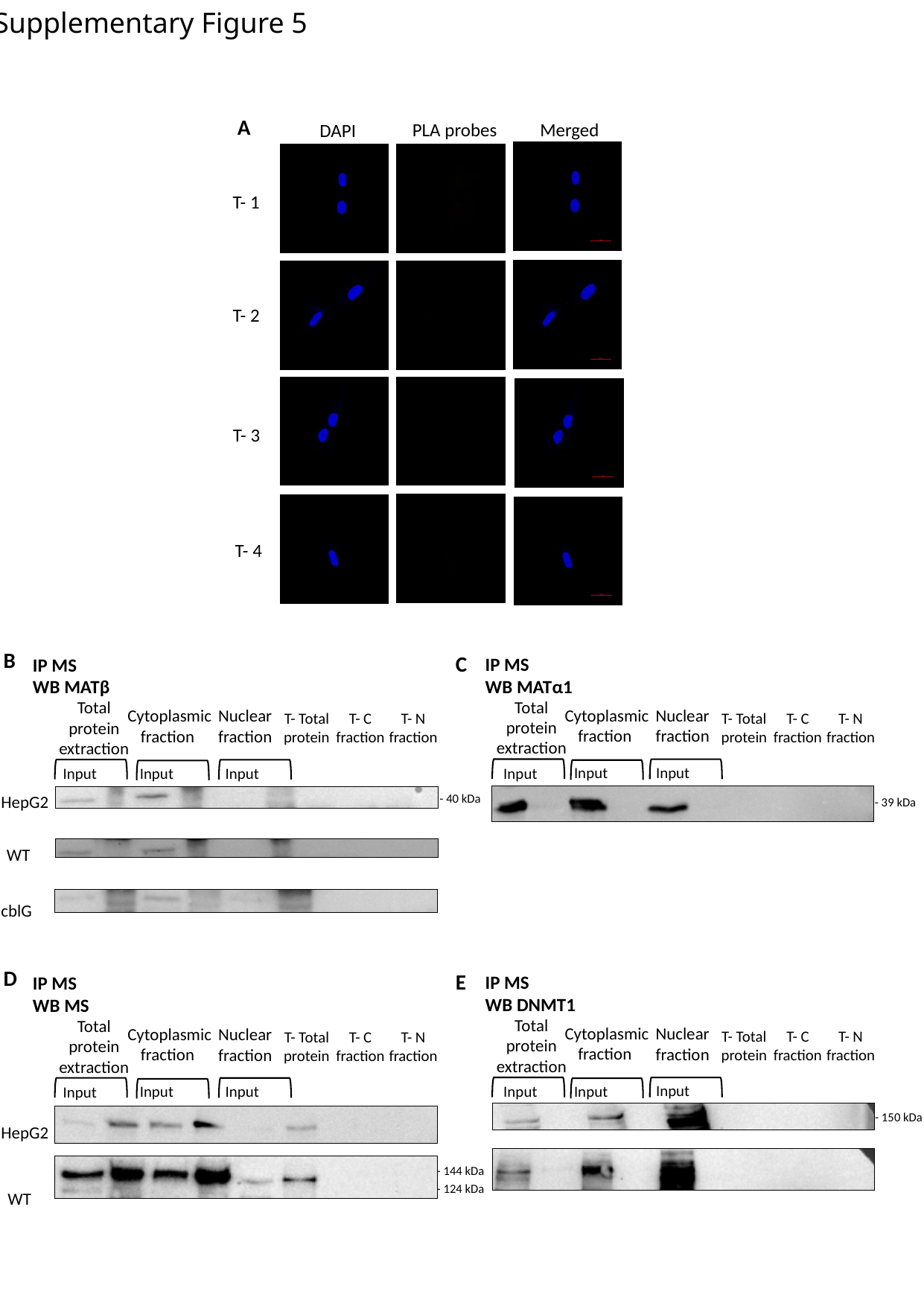

Supplementary Figure 5
A
PLA probes
Merged
DAPI
T- 1
T- 2
T- 3
T- 4
B
C
IP MS
WB MATα1
IP MS
WB MATβ
Total protein extraction
Total protein extraction
Cytoplasmic fraction
Nuclear fraction
Cytoplasmic fraction
Nuclear fraction
T- Total protein
T- C fraction
T- N fraction
T- Total protein
T- C fraction
T- N fraction
Input
Input
Input
Input
Input
Input
- 40 kDa
HepG2
- 39 kDa
WT
cblG
D
E
IP MS
WB DNMT1
IP MS
WB MS
Total protein extraction
Total protein extraction
Cytoplasmic fraction
Nuclear fraction
Cytoplasmic fraction
Nuclear fraction
T- Total protein
T- C fraction
T- N fraction
T- Total protein
T- C fraction
T- N fraction
Input
Input
Input
Input
Input
Input
- 150 kDa
HepG2
- 144 kDa
- 124 kDa
WT
