# Supplementary material for: Methionine Synthase Interacts With the Methionine Adenosyl‐Transferase MATα2 and the DNA Methyltransferase DNMT3b in the Nucleus
Source: J Inherit Metab Dis. 2026 Jun 17;49(4):e70211. doi: 10.1002/jimd.70211 (PMC13275206; doi:10.1002/jimd.70211)
Supplement: Supplementary file 3 — Figure S3: Nuclear location of MS and MS‐GFP plasmids il HEK293T cells and explication of nuclear MS location. (A) Control of immunofluorescence staining; AlexaFluor 488: Green staining. (B) Immunofluorescence staining of MS in HEK293T. Cells were stained with anti‐MS antibody and visualized by confocal microscopy (N = 3). Adjustments of individual colour channels were performed with Photoshop. (C) Expression of methionine synthase isoforms (124 kDa and 144 kDa) fused with green fluorescent protein (GFP) in HEK293T cells visualized by confocal microscopy (N = 3). Adjustments of individual colour channels were performed with Photoshop. (D) Control of immunofluorescence staining in HEK293T cells transfected with empty plasmid. (E) Zoom of methionine synthase isoforms (124 kDa and 144 kDa) fused with green fluorescent protein (GFP) in HEK293T cells presented in (C). (F) EdU Click‐iTTM staining with immunofluorescence staining of MS in HepG2 cells, control (WT) and cblG fibroblasts. Cells were stained with EdU and anti‐MS antibody and visualized by confocal microscopy (N = 3). [file JIMD-49-0-s004.pptx]

## Slide 1
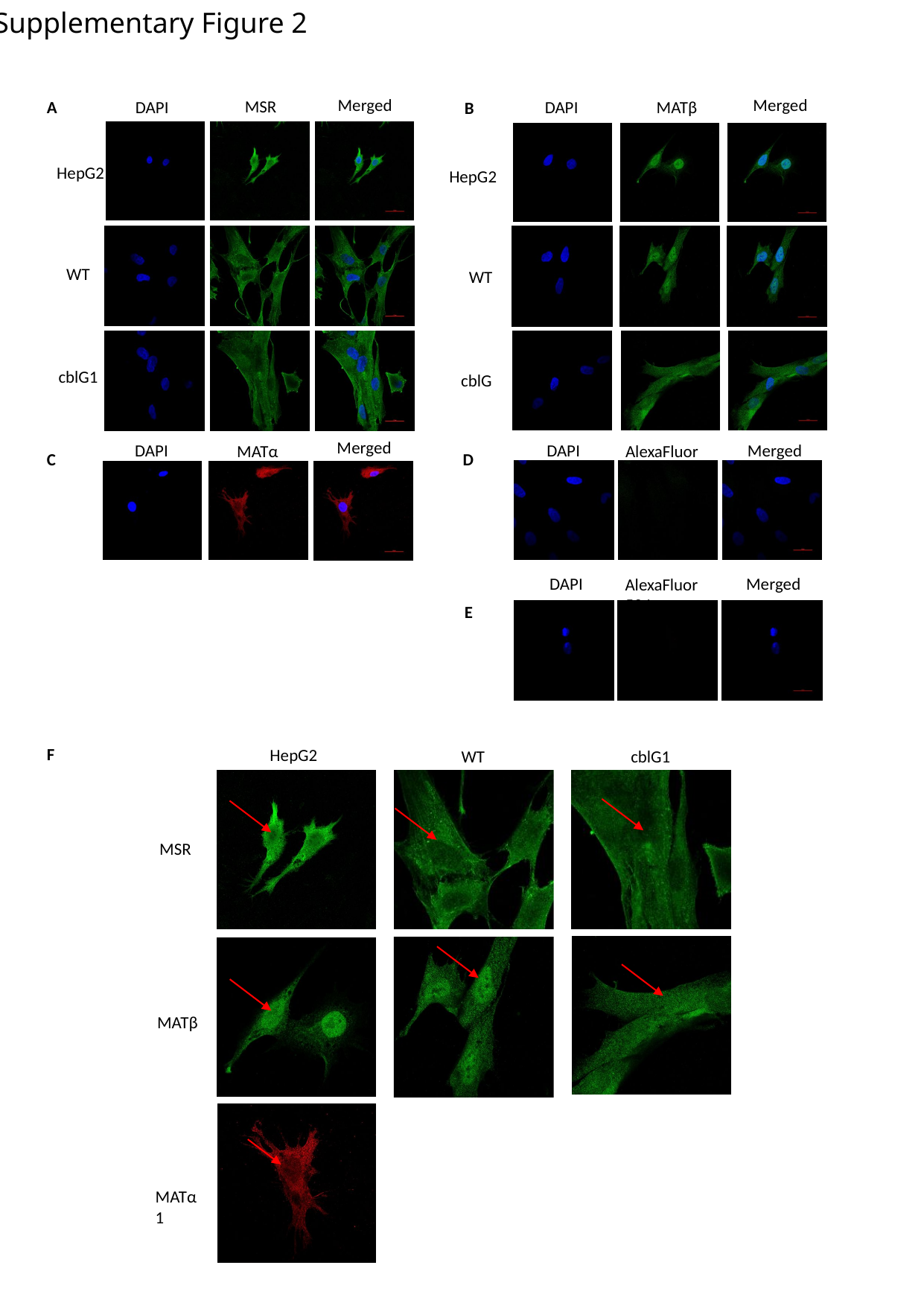

Supplementary Figure 2
Merged
MSR
DAPI
HepG2
WT
cblG1
Merged
DAPI
MATβ
HepG2
WT
cblG
A
B
Merged
DAPI
Merged
AlexaFluor 488
DAPI
MATα1
C
D
DAPI
Merged
AlexaFluor 594
E
F
HepG2
WT
cblG1
MSR
MATβ
MATα1
