# Supplementary material for: Methionine Synthase Interacts With the Methionine Adenosyl‐Transferase MATα2 and the DNA Methyltransferase DNMT3b in the Nucleus
Source: J Inherit Metab Dis. 2026 Jun 17;49(4):e70211. doi: 10.1002/jimd.70211 (PMC13275206; doi:10.1002/jimd.70211)
Supplement: Supplementary file 4 — Figure S4: Evidence of one‐carbon metabolism in cytoplasmic and nuclear fractions of HepG2 cells, control and cblG fibroblasts. (A–F) Metabolites of one‐carbon metabolism were measured by LC/MS–MS in cytoplasmic and nuclear fractions of HepG2 cells, control (WT) and cblG fibroblasts; methionine (A), S‐adenosyl‐L‐methionine (SAM) (B), S‐adenosyl‐homocysteine (SAH) (C), homocysteine (D), cystathionine (CTH) (E), and 5‐methyl‐tetrahydrofolate (5‐methyl‐THF) (F); Mean ± SEM., n = 3; **p < 0.01; ***p < 0.001; Two‐way ANOVA; GraphPad. (G) Schematic representation of incorporation of D4‐homocystein. (H–J) Measurement of D4‐homocystein (H), D4‐methionine (I), D4‐SAH (J) after in vitro incorporation of D4‐homocystein in cytoplasmic and nuclear fractions of HepG2 cells, control (WT) and cblG fibroblasts. [file JIMD-49-0-s002.pptx]

## Slide 1
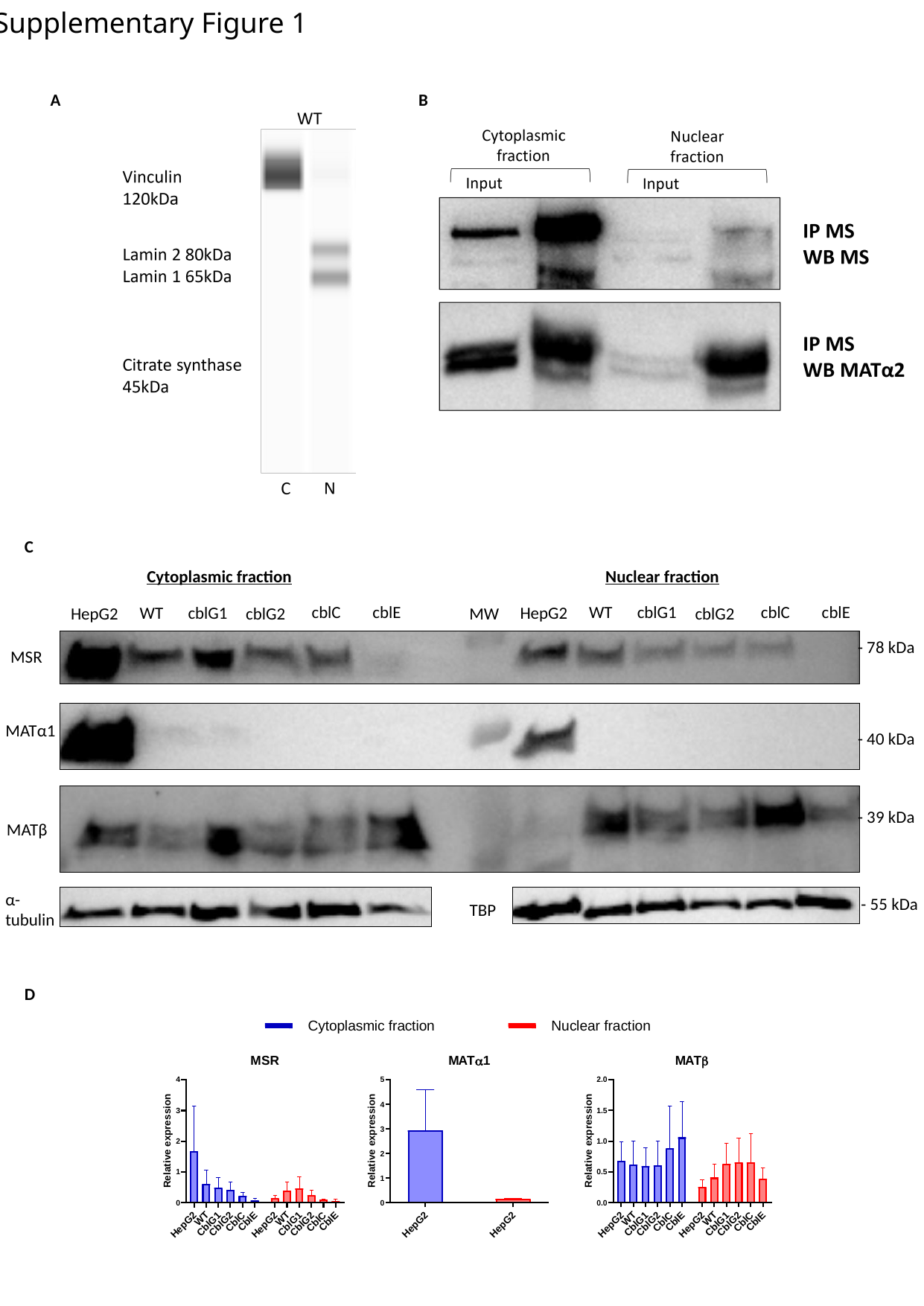

Supplementary Figure 1
B
A
C
Cytoplasmic fraction
Nuclear fraction
cblC
cblE
cblC
cblE
WT
cblG1
WT
cblG1
HepG2
HepG2
MW
cblG2
cblG2
- 78 kDa
MSR
MATα1
- 40 kDa
- 39 kDa
MATβ
α-tubulin
- 55 kDa
TBP
D
