# Supplementary figures and images for: Methionine Synthase Interacts With the Methionine Adenosyl‐Transferase MATα2 and the DNA Methyltransferase DNMT3b in the Nucleus
Source: J Inherit Metab Dis. 2026 Jun 17;49(4):e70211. doi: 10.1002/jimd.70211 (PMC13275206; doi:10.1002/jimd.70211)

## Slide 1
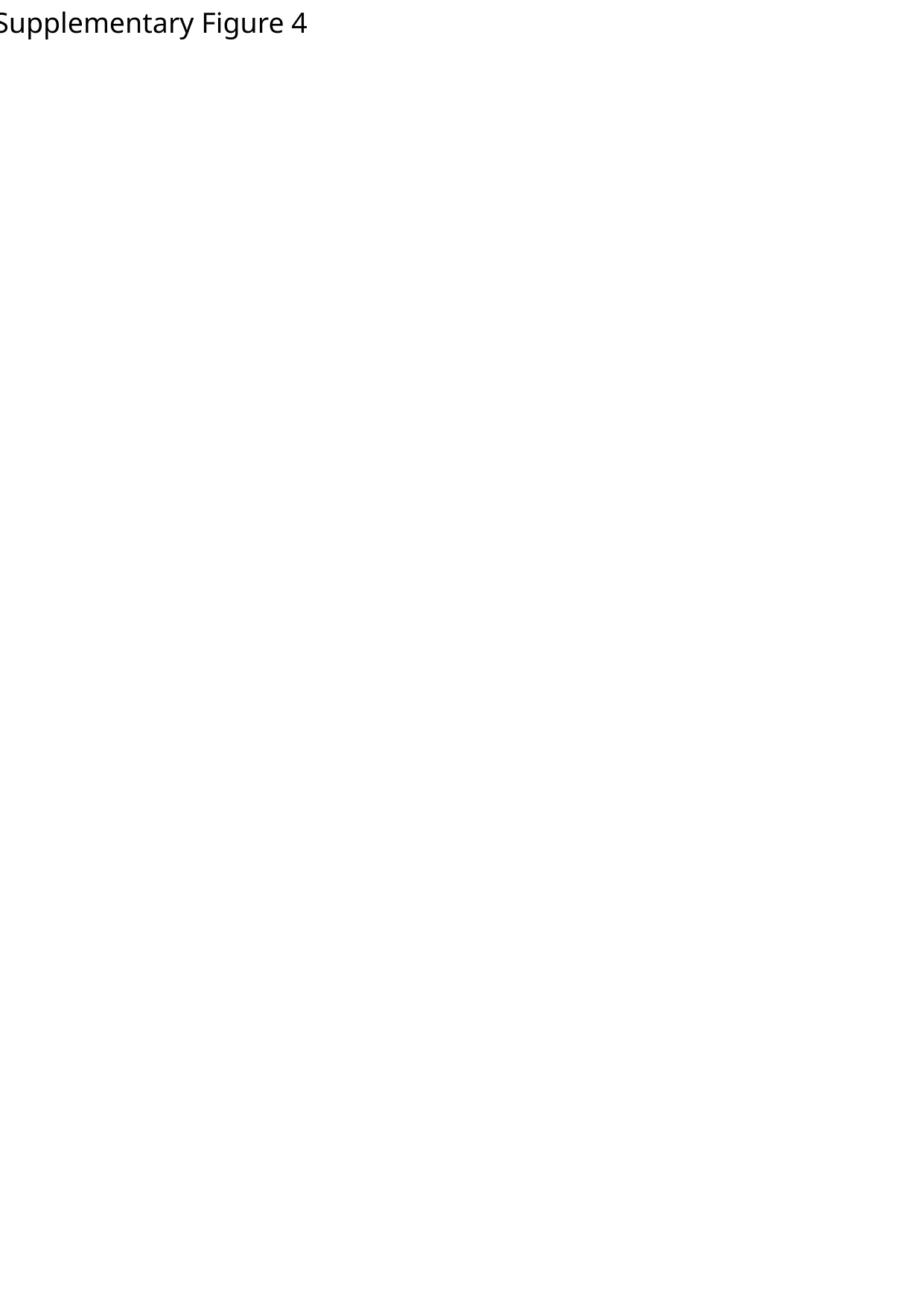

Supplementary Figure 4

Supplement: Supplementary file 5 — Figure S5: Evidence of absence of protein–protein interaction between methionine synthase (MS) and DNA methyltransferase 1 (DNMT1) in total protein extracts, cytoplasmic and nuclear fractions of HepG2 cells, control, and cblG fibroblasts. (A) Control of Duolink Proximity Ligation Assay; T‐1: Without primary antibodies and without PLA probes; T‐2: Without primary antibodies with PLA probes, T‐3: With anti‐MS antibody and with PLA probes; T‐4: With anti‐MATα2 antibody and with PLA probes. (B) Absence of interaction between MS and MATβ. Co‐immunoprecipitation assay was performed on HepG2 cells, control (WT), and cblG fibroblasts with antibody against methionine synthase (MS). Immunoprecipitated samples were subjected to western blot against MATβ (N = 4). C, cytoplasm; N, nucleus; T, control without antibody which realized immunoprecipitation; (C) Absence of interaction between MS and MATα1. Co‐immunoprecipitation assay was performed on HepG2 cells with antibody against methionine synthase (MS). Immunoprecipitated samples were subjected to western blot against MATα1 (N = 4). T‐: control without antibody which realized immunoprecipitation; C, cytoplasm and N, nucleus. (D) Validation of immunoprecipitation of MS in total protein extract, cytoplasmic and nuclear fractions of HepG2 cells and control (WT) fibroblasts as described in Figure 5A (N = 4). C, cytoplasm; N, nucleus; T, control without antibody which realized immunoprecipitation; (E) Absence of interaction between MS and DNMT1. Co‐immunoprecipitation assay was performed on total protein extract, cytoplasmic and nuclear fractions of HepG2 cells and control (WT) fibroblast cells with an antibody against methionine synthase (MS). Immunoprecipitated samples were subjected to western blot against DNMT1 (N = 3). C, cytoplasm; N, nucleus; T, control without antibody which realized immunoprecipitation. [file JIMD-49-0-s005.pptx]
